# Multi-Source Pathways of T Follicular Helper Cell Differentiation

**DOI:** 10.3389/fimmu.2021.621105

**Published:** 2021-02-25

**Authors:** Xiaoxue Ma, Shingo Nakayamada, Jun Wang

**Affiliations:** ^1^ Department of Pediatrics, The First Hospital of China Medical University, Shenyang, China; ^2^ Department of Microbiology & Immunology and Pediatrics, Dalhousie University, Halifax, NS, Canada; ^3^ Canadian Center for Vaccinology, IWK Health Centre, Halifax, NS, Canada; ^4^ First Department of Internal Medicine, School of Medicine, University of Occupational and Environmental Health, Japan, Kitakyushu, Japan

**Keywords:** T follicular helper cell, cytokine, signal transducer and activator of transcription, pathway, differentiation

## Abstract

T follicular helper (Tfh) cells participate in humoral immune by promoting inflammation and aiding B cells survival, proliferation, maturation, and generation autoantibodies. The plasticity of Tfh cells enables the immune system to adjust the direction of differentiation according to the degree of the immune response, regulate the germinal center (GC) response and maintain homeostasis. Tfh differentiation involves several signaling factors, including multiple cytokines, receptors, transcription factors and genes. The signal transducer and activator of transcription (STAT) family signaling pathways are crucial for Tfh formation. However, because of the multi-factorial and multi-stage features of Tfh differentiation, every STAT member plays a role in Tfh differentiation, but is not completely depended on. With the gradual recognition of different Tfh subsets (Tfh1, Tfh2, Tfh17), the process of Tfh differentiation can no longer be explained by straight-line derivation models. In this review, we summarize the roles of different STATs in mediating Tfh subsets, analyze the contributions of mutual restraint and cooperation among cytokine-STAT signals to terminal Tfh differentiation, and clarify the multi-source pathways of Tfh differentiation with a three-dimensional illustration.

## Introduction

Antibody production is reliant upon the formation of GCs in secondary lymphoid organs, where B cells undergo proliferation, differentiation, and somatic hypermutation aimed at producing high affinity antibodies. These processes occur dynamically after activation of B cells by Tfh cells (1). Tfh cells are a unique lineage of CD4^+^ T cells that express the master transcription factor B-cell lymphoma 6 (Bcl-6). They are phenotypically characterized by surface expression of C-X-C motif chemokine receptor 5 (CXCR5) and programmed cell death protein1 (PD-1), as well as production of interleukin (IL)-21 (2). While proper development of Tfh cells is critical for establishing strong humoral immunity to protect the host from microbial infections, abnormal Tfh immune responses have been associated with autoimmune diseases (1). Therefore, a clear understanding of Tfh differentiation is required for finding suitable targets to control the accumulation and activity of Tfh cells.

Tfh differentiation has been studied for more than a decade and the increasing discovery of factors relevant to the process of Tfh differentiation has raised more questions than answers. Therefore, dynamically observing the interactions between various signals will aid in our understanding of the processes of Tfh differentiation. In this review, we focus on the roles of cytokine-STAT signaling in regulating the multi-stage processes of Tfh formation and how multiple signals restrict and cooperate with each other to promote Tfh differentiation.

## Differentiation of Tfh Cells Is Not a Straight-Line

It is well known that Tfh cells are a fluid subpopulation. The differentiation of Tfh cells is multi-staged and multi-factorial. Thus, there is no single event that easily defines Tfh differentiation. Currently, a multi-stage differentiation model has been established to describe three essential processes of canonical Tfh differentiation, including the dendritic cell (DC) priming stage, T-B cell interaction stage and GC stage (3). However, it has been increasingly recognized that Tfh differentiation is more complex than just three stages. Precursor Tfh (pre-Tfh) cells share a common developmental program with Th1, Th2, or Th17 cells and the direction of polarization depends upon the dominant cytokine milieu (4). Due to the flexibility of T helper (Th) cells, Tfh cells express major regulator Bcl-6 and can express major regulators of other lineages, such as T box factor (T-bet), GATA binding protein 3 (GATA3), or retineic-acid-receptor-related orphan nuclear receptor gamma (RORT) (5). These dual transcriptional regulator-expressing cells are defined as subsets of Tfh cells named Tfh1, Tfh2 and Tfh17, respectively. Recently, T follicular regulatory (Tfr) cells have been identified as a new subset of T regulatory (Treg) cells that co-express Bcl-6 and forkhead box P3 (Foxp3). Tfr cells are able to access B-cell follicles and inhibit the TfhB cell response in GCs (6), which is indispensable for Tfh differentiation. Many reports have shown that alterations in the proportions of Tfh subsets and Tfr cells are associated with the pathogenesis of autoimmune and infectious diseases (7,11). The functional heterogeneity of Tfh cells suggests that multiple sources may contribute to the formation of terminally differentiated Tfh cells, which have developed the characteristics of their respective subsets and maintain the capacity for repolarization.

## Functional Diversity of Tfh Cells

Knowledge of the multi-source pathways of Tfh differentiation contributes to the understanding of the heterogeneity of phenotype and function of Tfh cells. Similar to T effector cells, the dominant cytokine milieu at the earliest stage of differentiation determines the fate of pre-Tfh cells. These fates determine the roles of terminal Tfh cells in the immune response. The Tfh1 differentiation pathway can be initiated by type 1 responses in which STAT1/4-activating cytokines expand, such as viral infection, vaccination, or some autoimmune diseases (9, 10, 12, 13). Tfh1-source subsets produce IFN- and IL-21 and lead to isotype switching of GC B cells to induce murine IgG_2_ or human IgG_1_ (14). Type 2 infections, allergic diseases or autoimmune diseases such as IgG_4_-related disease can initiate Tfh2 differentiation (8, 15, 16). Tfh2-source subsets produce IL-4 and IL-21 to support induction of either murine IgG_1_ or human IgG_4_ and IgE by GC B cells (14). Tfh17-source subsets induce IgA production (14). An increase in circulating Tfh17 cells can be found in patients with immunoglobulin A vasculitis, as well as a variety of immune diseases which are often accompanied by an increase in the Tfh2 subset (8, 10, 17).

## Cytokine-STAT Signaling Pathways Acting as Environment Sensors in T Cells

**Figure 1:**
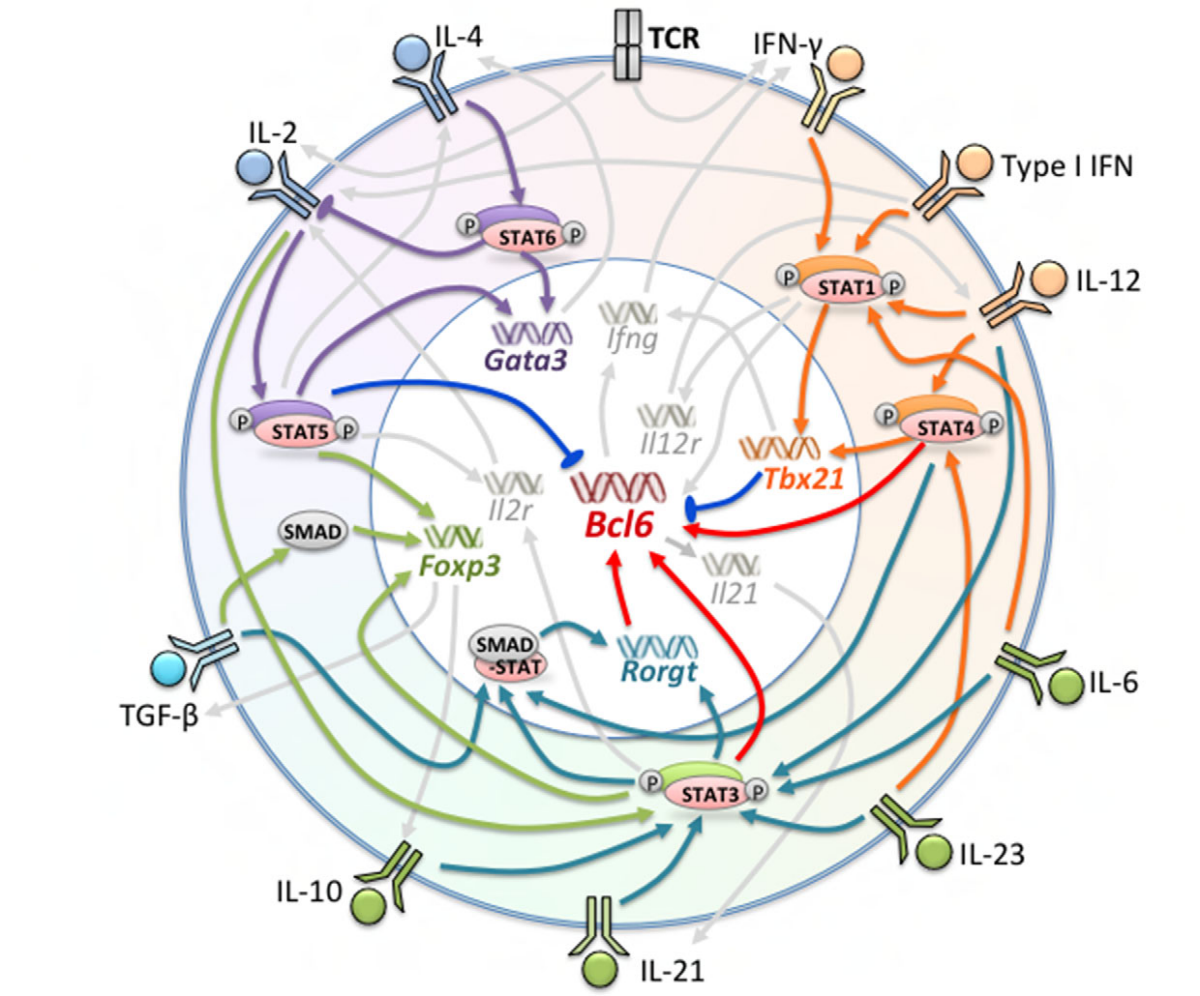
Factors Involved in the Generation of Tfh and Tfh Subsets. Multiple cytokines, receptors, and transcription factors are involved in the differentiation of Tfh cells. The orange, green, and purple regions represent factors leading to the polarization of Tfh1, Tfh17 and Tfh2 cells, respectively. Red arrows indicate promotion of Bcl-6 expression; blue arrows indicate suppression of Bcl-6 expression; orange, dark green, purple, and light green arrows indicate major regulated factors of Tfh1, Tfh17, Tfh2, and Tfr, respectively.

Molecular signals including cytokines, surface receptors, and transcription factors, are crucial regulators of Tfh differentiation at every stage. Cytokine signaling is a type of ubiquitous and indispensable molecular signal in immune cells and is critical for cell survival, proliferation, apoptosis, and differentiation (18). Many molecular signals have been shown to be directly or indirectly involved, positively or negatively regulating Tfh formation and maintenance (**Figure 1**).

Many cytokines act through the JAK-STAT signaling pathways to exert their effects on Tfh differentiation. Binding of cytokines to theirs receptors and receptor dimerization leads to the activation of JAKs, which phosphorylate the receptor tails and induce docking of STATs to the receptors. Phosphorylated STATs dimerize and move into the nucleus, activating the transcription of target genes (19, 20). The STAT family shares a characteristic protein domain structure and consists of seven members-STAT1, STAT2, STAT3, STAT4, STAT5a, STAT5b, and STAT6 (21). Each STAT can be activated by multiple cytokines. For example, STAT3, which is predominantly activated by IL-6 and IL-21, can also be activated by IL-23, IL-12, and IL-10 (22, 23). Similarly, most cytokines can activate more than one STAT member. For instance, type I IFN is able to activate almost all of the STATs (19, 24). Intriguingly, it seems that different STAT signals activated by one single cytokine can be competitive, whereas the same STAT signal activated by different cytokines can compensate for one another. For example, IL-6 elicits a strong STAT3 response coupled to a weaker STAT1 response. In the absence of STAT3, IL-6 enhances activation of STAT1 (25). Addition of STAT3-activating cytokine IL-23 to IL-6 generates a more serious Th17-mediated autoimmunity (26).

## The Roles of STAT Factors in Early Tfh Differentiation

### STAT1 and STAT4 Initiate the Tfh1 Pathway

#### STAT1 as a Pioneer

STAT1 is a well known Th1-polarizing signal (27). In a recent fate-mapping analysis, almost all IFN--producing Tfh cells previously expressed T-bet (28). Excessive IFN- signaling and IFN- receptors result in pathological accumulation of Tfh cells and GC formation (29). During acute viral infection, T-bet is required for the expansion and maintenance of Tfh1 cells (30). In addition, other STAT1-activating cytokines also promote Tfh differentiation. Type I IFN and IL-6 are able to induce Bcl-6 expression and early Tfh differentiation *via* activation of STAT1 (31, 32). The studies of STAT1-mediated Tfh differentiation have mainly been performed in mice. In humans, the impact of STAT1 on Tfh cells has been visualized mainly through IL-12-mediated co-activation of STAT1 and STAT4 (10, 33). Interestingly, STAT1 is a regulator of IL-12 receptor (IL-12R) expression. IL-12R is not expressed on human naive CD4^+^ T cells until it is induced by IFN--STAT1 signaling derived from TCR-stimulation (10). Analogous processes were also verified in mice (34).

However, STAT1 seems to be indispensable only at the early stage of Tfh differentiation. IFN--producing Tfh cells were absent in T-bet-deficient mice, but were present in the mice with T-bet deletion at later stages of differentiation (28). Moreover, Type I IFN-STAT1 was an inefficient inducer of IL-21 production (31). In humans, T-bet promotes CXCR5 expression but diminishes the ability of Tfh cells to provide help to B cells (35). In summary, STAT1 acts as a pioneer for initial induction of the Tfh1 pathway, but additional signals are needed for subsequent differentiation.

#### STAT4 Cooperation With STAT1

STAT4 is activated predominantly by IL-12 and to a lesser extent by IL-23 and Type I IFN (36). In humans, one study showed that DCs induced IL-21-producing Tfh differentiation through production of IL-12 (37). IL-12 was able to promote sustained expression of CXCR5 and Bcl-6 in activated CD4^+^ T cells independent of IFN- or T-bet (38). Patients with IL-12R_1_ deficiency had fewer Tfh cells, memory B cells and GCs (39, 40). In mice, ChIP experiments indicated that expression of the *il21* and *bcl6* genes was promoted by IL-12-mediated STAT4 signaling (41, 42).

Similar as STAT1, STAT4 can induce the Th1 phenotype and thus may induce Tfh1 cell differentiation (27). It has been shown that production of IL-21 and IFN- is strongly inhibited in STAT4-knockdown CD4^+^ T cells (37). Further study confirmed that IL-12 not only activated STAT4, but also activated STAT1 independent of exogenous IFN-, resulting in the expansion of Tfh1 cells (10). However, although IL-12 modulated the differentiation of IL-21^+^IFN-^+^Tfh1 cells from naive CD4^+^ T cells *via* both STAT1 and STAT4, only STAT4 was indispensable if the induction of differentiation was initiated from memory cells (33). STAT1-deficiency did not reduce IL-12-induced Tfh cells (40).

However, singular STAT4 signaling plays only a transient role relative to the entire Tfh differentiation process. One study showed that STAT4-induced T-bet repressed *bcl6* gene expression and thus decreased the expansion of Tfh cells and attenuated Tfh-related functions. Impaired generation of Tfh cells resulted in differentiation toward the Th1 phenotype (41). Consequently, participation of additional signals is required to rescue STAT1/4-induced Th1 polarization and thus complete subsequent differentiation.

### Distinct Roles of STAT5 and STAT6 in the Tfh2 Pathway

IL-4-STAT6 signaling is able to promote the expression of GATA3 and is thought to be crucial for Th2 differentiation (27, 43, 44). Presently, studies of Tfh2 differentiation have been performed mainly in mice. It has been shown that during helminth infection, IL-4-producing CD4^+^ T cells in reactive lymph nodes have the phenotypic characteristics of Tfh cells, suggesting that these IL-4-producing cells are indeed Tfh cells (45). Further study demonstrated that Bcl-6^+^GATA3^+^Tfh2 cells in lymph nodes are derived from Th2 cells (46). IL-2-STAT5 signaling also promoted Th2 phenotypic characteristics by up-regulating the expression of IL-4R and the production of IL-4 (47,50).

The induction of Tfh2 differentiation by IL-2-STAT5 seems paradoxical, since STAT5 contributes to Th2 gene expression but represses Tfh development (51, 52). In contrast, IL-4-STAT6 signaling can promote Tfh2 differentiation. It has been reported that IL-4 selectively suppresses IL-2-STAT5 and IL-2R expression (53), which benefits Tfh differentiation in theory. Thus far, it is known that STAT6, in cooperation with STAT3, contributes to the capacity of Bcl-6^+^GATA3^+^Tfh2 cells to provide B-cell help (54). It has also been shown that Batf, in cooperating with STAT3 and STAT6, increases the production of IL-4 in Tfh cells (55). However, it seems that STAT3 should not be activated prematurely, since STAT3-deficient Tfh cells overexpressed both Bcl-6, GATA3, and IL-4, which suggests that the intrinsic effects of STAT3 on Tfh2 cells at the early stage of differentiation are suppressive (56).

### STAT3 Participates in the Differentiation of Tfh17 Cells

#### Controversial Functions of IL-6/IL-21-STAT3

IL-6/IL-21-STAT3 signaling is generally considered as the most desirable factors for Tfh formation, although there is much controversy (14). At the present, most conclusions supporting this theory have been derived from *in vivo* experiments. It has been reported that lack of IL-6 and/or IL-21 reduces the formation of Tfh cells and GCs (57, 58). However, another study found that absence of IL-21 influenced GC B cells but failed to abrogate Tfh formation (59). Further study clarified that the effect of IL-21 on GCs was a result of direct action upon B cells independent of Tfh cells. Tfh cells were formed but decreased faster in absence of IL-21 (60). However, other studies have shown that neither IL-21 nor IL-6 was required for Bcl-6^+^Tfh differentiation (61). IL-6R deletion in T cells did not affect the accumulation of Tfh cells (62).

In one *in vitro* study, purified murine naive CD4^+^ T cells did not express more Bcl-6 or CXCR5 in the presence of IL-6 or IL-21, although abundant production of IL-21 was found in culture environments (58). In humans, although STAT3 mutations in patients compromised the generation of Tfh cells, the expression of CXCR5 and Bcl-6 in STAT3-deficient CD4^+^ T cells *in vitro* was not impacted (40). Therefore, STAT3 alone seems to be insufficient for Tfh formation, especially in purified CD4^+^ T cells.

#### TGF- plus STAT3/STAT4 and the Tfh17 Phenotype

Although the role of STAT3 alone is undefined, transforming growth factor (TGF-) and STAT3/STAT4-activating cytokines in combination can promote human Tfh differentiation. Research has demonstrated that TGF- plus IL-12/IL-23 was the most effective in up-regulating the expression of CXCR5, Bcl-6 and IL-21, while other STAT3-activating cytokines synergistically enhanced Tfh differentiation (22). It is known that TGF-, IL-1, IL-6, and IL-23 in combination can increase the expression of RORT and IL-17 (63,65). Therefore, it has been inferred that TGF- plus STAT3/STAT4 signaling can induce Tfh17 differentiation.

TGF- promotes Treg differentiation in the presence of IL-2 (66). It has also been shown that TGF- can insulate pre-Tfh cells from IL-2-delivered mTOR signaling, thereby improving Tfh formation (67). Th17 differentiation is promoted by TGF- through the up-regulation of IL-17A and down-regulation of IFN- production (68). Meanwhile, IL-23-induced STAT3 can strongly bind the *Rorc* gene locus to promote Th17 differentiation (65). STAT4 can prevent Th17 polarization by inducing Tfh1 differentiation. IL-6 is beneficial for both Th1 and Th17 differentiation, and it is also able to inhibit the generation of Treg cells (69). Therefore, we speculate that the effects of the cocktail stimulation result from the mutual restriction of each factor.

### IL-2-STAT5 Signaling Acts as a Rheostat by Regulating Tfr Cells

IL-2-STAT5 signaling is thought to negatively regulate Tfh differentiation by repressing Bcl-6 expression (51, 52). Low-dose IL-2 reduced disease activity in lupus patients by suppressing the expansion of Tfh cells (70). However, a recent study showed that IL-2-STAT5 seems to fine-tune the differentiation of Tfh, Treg, Tfr cells according to its concentration during an immune response. During influenza infection, high IL-2 concentrations at the peak of infection prevented the differentiation of Tfr cells from Treg cells by inhibiting Bcl-6 expression to permit GC responses (71). Meanwhile, Treg differentiation is enhanced, and these cells highly express IL-2R (CD25), which gradually consumes IL-2 in the milieu. With the depletion of IL-2, the suppression of Tfr cells alleviated and GC responses are inhibited (71, 72).

It has been reported that Tfr cells can differentiate from Treg cells (73). However, our recent study showed that IL-2 induced the transformation of human Tfh cells into Tfr-like cells. ChIP experiments showed that while promoting *Foxp3* expression, IL-2 also maintained *Bcl6* gene expression *via* activation of STAT3 (11). Therefore, IL-2-STAT5 should be considered a regulator of Tfr differentiation rather than a pure inhibitor. These results suggest that the role of IL-2 may differ between humans and mice.

## Restriction Among STATs Contributes to the Subsequent Differentiation of Tfh Cells

### STAT3 Remedies Over-polarization of Tfh1 by Restricting STAT1

One study showed that although STAT3 had no direct impact on the expression of Bcl-6 and CXCR5, IL-6-STAT3 signaling prevented Th1 polarization induced by STAT1, thus indirectly promoting Tfh differentiation by synergizing with STAT1 (32). In the absence of STAT3, both IL-6 and IL-21 can prolong the activation of STAT1 and increase the expression of IFN--inducible genes (25, 74). It has also been reported that IL-6 enhanced the responses of Tfh cells only during late stages of chronic viral infection (75). Furthermore, IFN-^+^IL-21^+^CXCR5^+^cells and IFN-^+^ GC Tfh cells expanded in IL-10-deficient mice (23), which suggests that STAT3 may promotes subsequent differentiation of Tfh cells from Tfh1 cells. Consequently, delayed STAT3-activation relative to STAT1 seems to be beneficial for terminally differentiated Tfh cells. This speculation is also consistent with the actual developmental process of Tfh cells, as STAT1/STAT4-initiated Tfh1 can produce IL-21. IL-21-STAT3 signaling gradually increases and induces further Tfh formation.

### STAT5 Regulates the Development of Tfh Cells and Subsets

One study demonstrated that STAT5 binding to the *Bcl6* promoter increases in high IL-2 conditions, while STAT3 binding decreases, thereby repressing Bcl6 expression (76). However, although pre-Tfh cells are IL-2-producing cells (77), large amounts of autocrine IL-2 did not impact Tfh differentiation, due to the IL-2 responsiveness regulated by IL-6-STAT3 (78). Another recent study showed that IL-6-STAT3 prevented the association of STAT5 with the *Il2rb* locus and repressed IL-2R (CD122) expression, which interrupted the IL-2-STAT5-IL-2R inhibitory feedback loops and allowed for sustained development of Tfh cells (78). In contrast, Type I IFN is able to induce the expression of IL-2R, which leads to STAT5 binding at the expense of STAT3, leading to a reduction in Tfh cell differentiation (79).

In addition, it has been reported that IL-2-STAT5 can also induce the expression of IL-12R. Similar to the promoting of Tfh2 differentiation *via* inducing IL-4R expression, IL-2-STAT5 involved the differentiation of Tfh1 cells *via* regulating IL-12R expression (80). Therefore, modulation of cytokine receptors by IL-2-STAT5 broadly regulates differentiation into T helper cell lineages.

## Discussion

**Figure 2:**
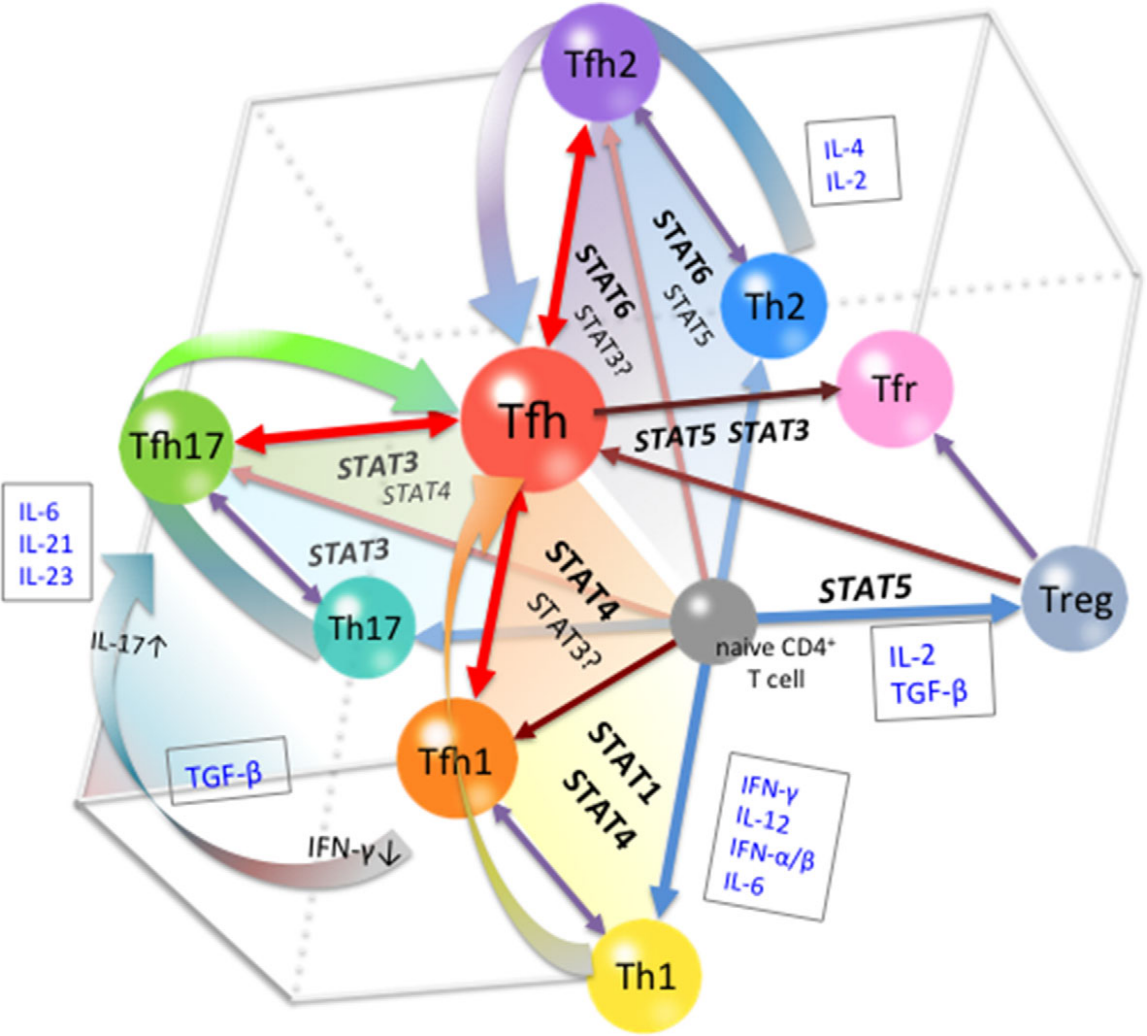
Cytokine-STAT-mediated Multi-source Pathways of Tfh Differentiation. The pathway of differentiation is determined by the type of stimulus to which the pre-Tfh cells are exposed to during the initial stage of differentiation. STAT1/STAT4-activating cytokines, such as Type I IFN and IL-12, guide cells toward the Tfh1 differentiation pathway; STAT6/STAT5-activating cytokines, such as IL-4 and IL-2, guide cells toward the Tfh2 differentiation pathway; and STAT3-activating cytokines, such as IL-21, IL-6, and IL-23, guide cells toward the Tfh17 differentiation pathway. At subsequent stages, restriction among STATs limits the polarization of Tfh subsets, leading to the completion of Tfh cell differentiation.

In conclusion, the differentiation of Tfh cells is a multi-stage and multi-source process. At the early stage of differentiation, environmental signals are indispensable in conferring the heterogeneity of Tfh phenotypes. Cytokines are the major factors that determine fate commitment, mainly through the activation and regulation of STATs (**Figure 2**). At subsequent stages, the restriction and cooperation of multiple STAT signals can prevent overpolarization of any Tfh subsets, thereby maintaining the expression of Bcl-6 in Tfh cells. The plasticity of the Tfh differentiation system is important for the control of adaptive immunity and homeostasis. In the overall Tfh differentiation process, the role of any factor is not absolutely positive or negative, but depends on the state of the cells, the stage of differentiation, and the polarity of the environment. In this review, we used STAT family members to illustrate the mechanisms of multi-stage and multi-source differentiation of Tfh cells. However, it should be noted that STATs are not the only transcription factors involved in the regulation of Tfh differentiation, and the important roles of other types of transcription factors should not be neglected. The detailed regulation of Tfh differentiation at each stage and the development of each subset need further study.

## Author Contributions

XM and SN created the research concept, designed and wrote the manuscript. JW was involved in the modification of the manuscript. All authors contributed to the article and approved the submitted version.

## Funding

This work was supported by the National Natural Science Foundation of China (grant 81901658); Infection, Immunity, Inflammation & Vaccinology (I3V) Dalhousie Medical Research Foundation (DMRF) Dr. David H. Hubel Postdoctoral Fellowship program; Nova Scotia COVID-19 Health Research Coalition (PI) Canada; Canadian Institutes of Health Research, 201803PJT-159700 to JW.

## Conflict of Interest

SN has received consulting fees, speaking fees, and/or honoraria from Bristol-Myers, GlaxoSmithKline, Pfizer, Chugai, Astellas, Sanofi, Amgen, Asahi-kasei (less than $10,000 each) and has received research grants from Mitsubishi-Tanabe, Takeda, Novartis and MSD.

The remaining author declares that the research was conducted in the absence of any commercial or financial relationships that could be construed as a potential conflict of interest.
