# Corrigendum: Multi-Source Pathways of T Follicular Helper Cell Differentiation

**DOI:** 10.3389/fimmu.2021.690815

**Published:** 2021-05-07

**Authors:** Xiaoxue Ma, Shingo Nakayamada, Jun Wang

**Affiliations:** ^1^ Department of Pediatrics, The First Hospital of China Medical University, Shenyang, China; ^2^ Department of Microbiology & Immunology and Pediatrics, Dalhousie University, Halifax, NS, Canada; ^3^ Canadian Center for Vaccinology, IWK Health Centre, Halifax, NS, Canada; ^4^ First Department of Internal Medicine, School of Medicine, University of Occupational and Environmental Health, Japan, Kitakyushu, Japan

**Keywords:** T follicular helper cell, cytokine, signal transducer and activator of transcription, pathway, differentiation

In the original article, we neglected to include the funder Canadian Institutes of Health Research, 201803PJT-159700 to JW.

In the original article, there was an error in the affiliation**s** for Xiaoxue Ma. As well as having affiliations 1 and 2, XM should also have Canadian Center for Vaccinology, IWK Health Centre, Halifax, NS, Canada.

In the original article, Jun Wang was not included as an author. The corrected Author Contributions Statement appears below.

XM and SN created the research concept, designed and wrote the manuscript. JW was involved in the modification of the manuscript. All authors contributed to the article and approved the submitted version.

The authors apologize for these errors and state that this does not change the scientific conclusions of the article in any way. The original article has been updated.

